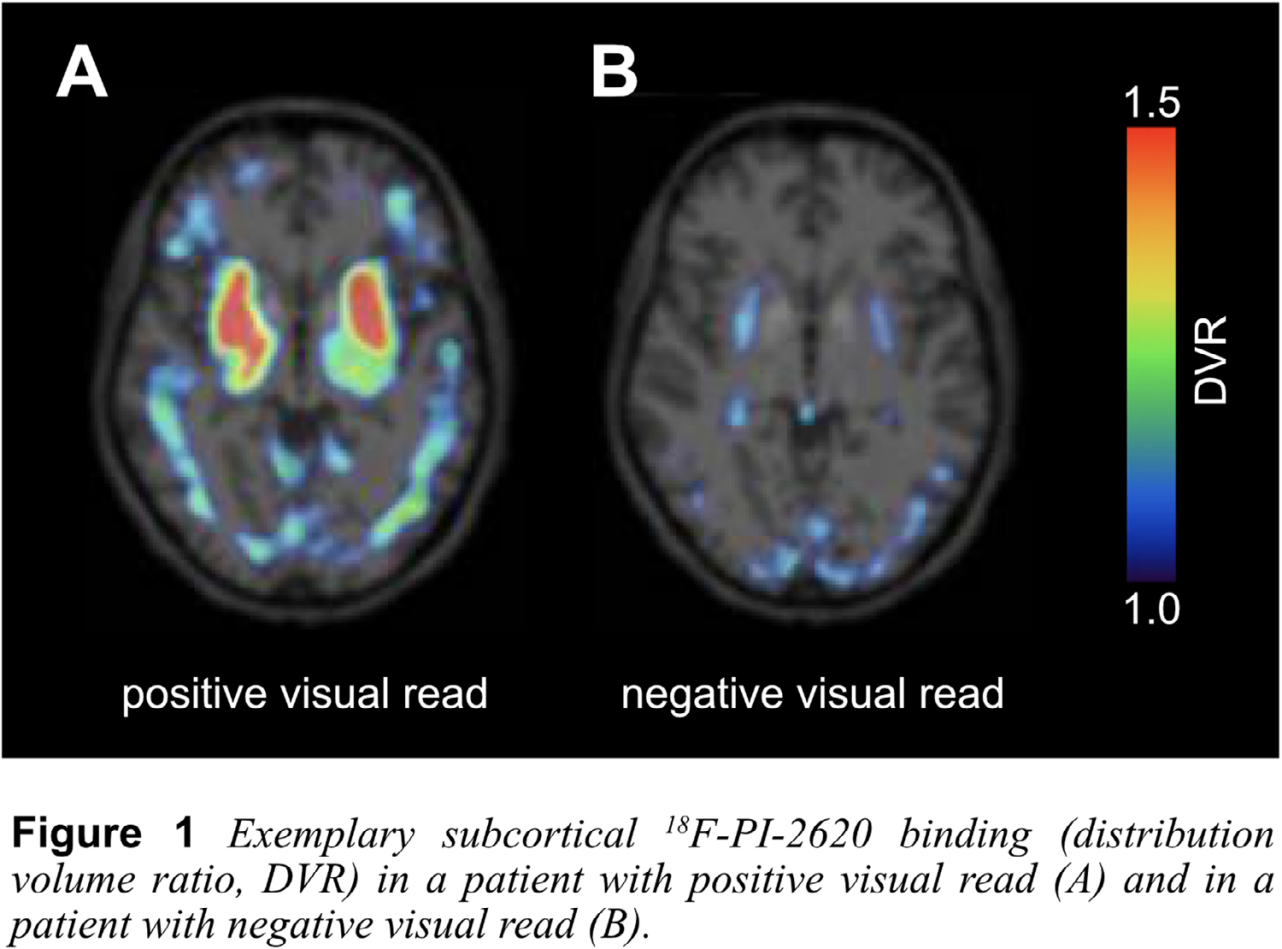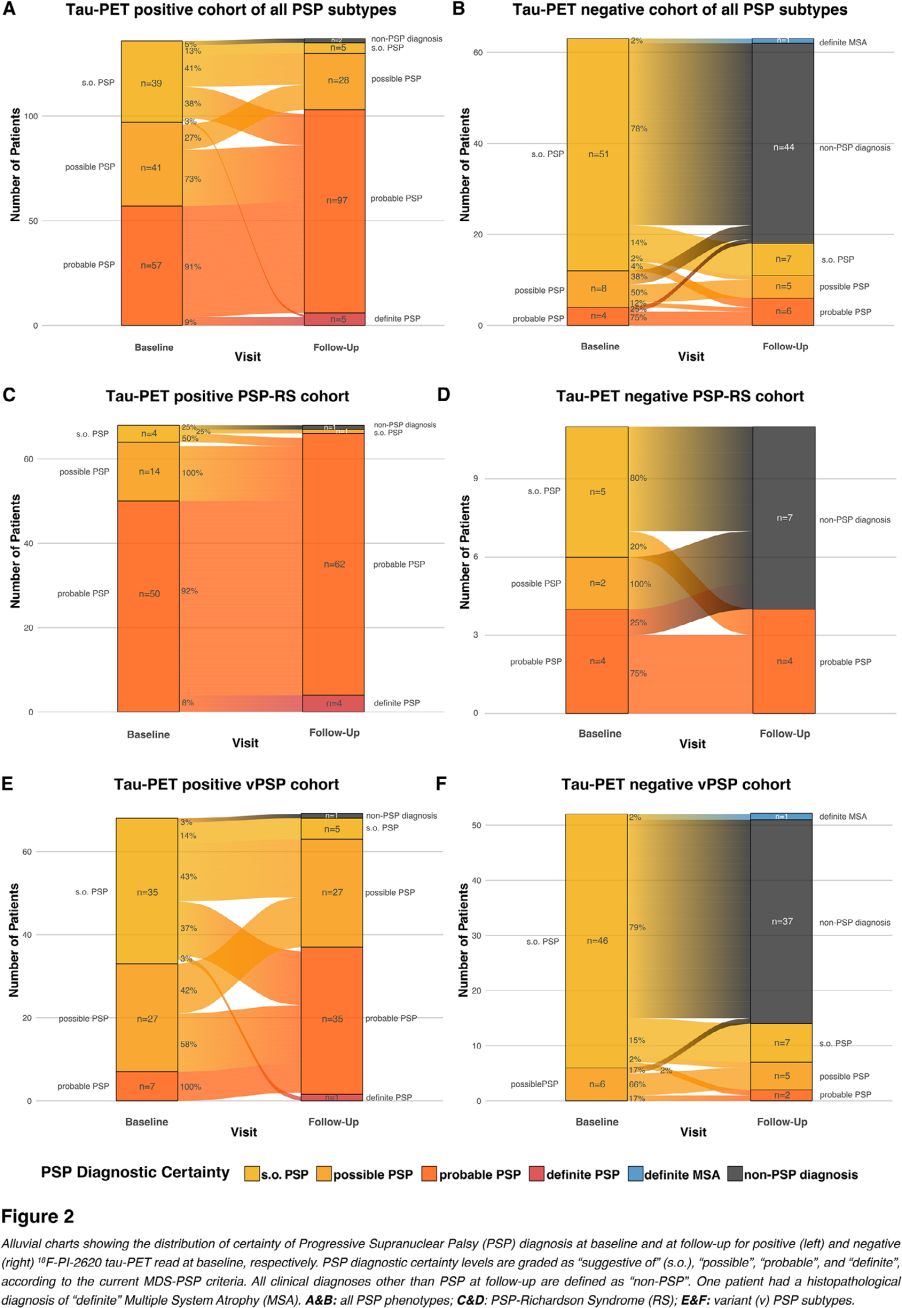# Clinical value of ^18^F‐PI2620‐PET in the diagnostic workup of patients with suspected Progressive Supranuclear Palsy

**DOI:** 10.1002/alz70856_102121

**Published:** 2025-12-25

**Authors:** Carla Palleis, Nicolai Franzmeier, Johannes Gnörich, Alexander Jäck, Alexander M Bernhardt, Sabrina Katzdobler, Urban M Fietzek, Endy Weidinger, Lukas Frontzkowski, Sebastian Roemer‐Cassiano, Andreas Zwergal, Henryk Barthel, Osama Sabri, Johannes Levin, Günter U Höglinger, Matthias Brendel

**Affiliations:** ^1^ German Center for Neurodegenerative Diseases (DZNE), Munich, Germany; ^2^ University Hospital, LMU Munich, Munich, Germany; ^3^ Munich Cluster for Systems Neurology (SyNergy), Munich, Germany; ^4^ University of Gothenburg, The Sahlgrenska Academy, Institute of Neuroscience and Physiology, Psychiatry and Neurochemistry, Gothenburg, Sweden; ^5^ Institute for Stroke and Dementia Research (ISD), University Hospital, LMU Munich, Munich, Bavaria, Germany; ^6^ Munich Cluster for Systems Neurology (SyNergy), Munich, Bavaria, Germany; ^7^ Institute for Stroke and Dementia Research (ISD), LMU University Hospital, LMU, Munich, Bavaria, Germany; ^8^ University Hospital, LMU Munich, Munich, Bavaria, Germany; ^9^ German Center for Neurodegenerative Diseases (DZNE), Munich, Bavaria, Germany; ^10^ Schön Klinik Schwabing, Munich, Bavaria, Germany; ^11^ Department of Neurology, University Hospital, LMU Munich, Munich, Bavaria, Germany; ^12^ Max Planck School of Cognition, Leipzig, Sachsen, Germany; ^13^ Department of Nuclear Medicine, University of Leipzig, Leipzig, Germany; ^14^ Leipzig University Medical Center, Leipzig, Germany; ^15^ Munich Cluster for Systems Neurology (SyNergy), Munich, Munich, Germany; ^16^ LMU University Hospital, Munich, Germany

## Abstract

**Background:**

Progressive Supranuclear Palsy (PSP) is a rapidly progressing 4‐repeat tauopathy, presenting with clinically heterogeneous phenotypes. Currently, diagnoses are based solely on clinical criteria but reliable diagnostic classification remains particularly challenging at early stages. ^18^F‐PI‐2620 tau‐PET is an evolving neuroimaging biomarker to capture 4‐repeat tau (4RT) deposits in vivo with clear diagnostic potential in research settings. To determine the added clinical value of ^18^F‐PI‐2620 tau‐PET in the diagnostic workup of PSP, we evaluated whether ^18^F‐PI‐2620‐assessed 4RT positivity (i.e. using the basal ganglia as a target readout) predicts subsequent increases of diagnostic certainty for PSP, indicative of 4RT pathology driving clinical progression.

**Method:**

We collected monocentric longitudinal data at the LMU Hospital in Munich, from a non‐randomized prospective cohort study between October 2018 and December 2024. Data collection included pre‐PET visits with routine clinical classification following the MDS criteria. In addition, we performed ^18^F‐PI‐2620 tau‐PET with dichotomous visual read assessments of 4RT pathology by an expert reader and collected clinical follow‐up data or autopsy information.

**Results:**

342 patients with a pre‐PET differential diagnosis of PSP were referred to ^18^F‐PI‐2620 tau‐PET in clinical routine. Of those, 200 patients (61.5% male, mean±sd age 69.2±8.3 years) had a post‐PET clinical follow‐up between 12‐24 months (mean±sd 17.1±4.2 months). 137 patients (68.5%) were rated 4RT‐positive at baseline (Figure 1). The distribution of certainty of PSP diagnosis at baseline and at follow‐up is displayed in Figure 2 (**A&B**: all PSP phenotypes; **C&D**: PSP‐Richardson Syndrome [RS]; **E&F**: variant PSP subtypes). Change to a non‐PSP diagnosis at follow‐up occurred in 23.5%, identified by a negative baseline tau‐PET in 95.5%. In contrast, 79% of tau‐PET‐positive patients with suggestive PSP progressed to a higher diagnostic certainty, 3% had histopathological confirmation of PSP diagnosis, 13% remained suggestive PSP, and 5% received a non‐PSP diagnosis at follow‐up.

**Conclusion:**

^18^F‐PI‐2620 tau‐PET can successfully identify patients that progress along expected 4RT clinical spectra. This supports ^18^F‐PI‐2620 tau‐PET as a 4RT biomarker, with the potential to facilitate early biomarker‐based diagnosis when clinical criteria may still lack sensitivity and specificity. This development can be transformative for clinical decision making, pre‐symptomatic identification of PSP and stratifying patients for disease modifying clinical trials.